# Allergen and Epitope Targets of Mouse-Specific T Cell Responses in Allergy and Asthma

**DOI:** 10.3389/fimmu.2018.00235

**Published:** 2018-02-13

**Authors:** Véronique Schulten, Luise Westernberg, Giovanni Birrueta, John Sidney, Sinu Paul, Paula Busse, Bjoern Peters, Alessandro Sette

**Affiliations:** ^1^La Jolla Institute for Allergy and Immunology, La Jolla, CA, United States; ^2^Division of Clinical Immunology, Icahn School of Medicine at Mount Sinai, New York, NY, United States; ^3^Department of Medicine, University of California San Diego, La Jolla, CA, United States

**Keywords:** mouse allergy, asthma, T cell epitope, Mus m 1, allergic rhinitis

## Abstract

Mouse allergy has become increasingly common, mainly affecting laboratory workers and inner-city households. To date, only one major allergen, namely Mus m 1, has been described. We sought to identify T cell targets in mouse allergic patients. PBMC from allergic donors were expanded with either murine urine or epithelial extract and subsequently screened for cytokine production (IL-5 and IFNγ) in response to overlapping peptides spanning the entire Mus m 1 sequence, peptides from various Mus m 1 isoforms [major urinary proteins (MUPs)], peptides from mouse orthologs of known allergens from other mammalian species and peptides from proteins identified by immunoproteomic analysis of IgE/IgG immunoblots of mouse urine and epithelial extracts. This approach let to the identification of 106 non-redundant T cell epitopes derived from 35 antigens. Three major T cell-activating regions were defined in Mus m 1 alone. Moreover, our data show that immunodominant epitopes were largely shared between Mus m 1 and other MUPs even from different species, suggesting that sequence conservation in different allergens is a determinant for immunodominance. We further identified several novel mouse T cell antigens based on their homology to known mammalian allergens. Analysis of cohort-specific T cell responses revealed that rhinitis and asthmatic patients recognized different epitope repertoires. Epitopes defined herein can be formulated into an epitope “megapool” used to diagnose mouse allergy and study mouse-specific T cell responses directly *ex vivo*. This analysis of T cell epitopes provides a good basis for future studies to increase our understanding of the immunopathology associated with MO-allergy and asthma.

## Significance Statement

Allergic Sensitization to mouse is a strong risk factor for the development of asthmatic disease, yet little is known about the allergic T cell response to mouse. We have identified 106 non-redundant epitopes from 35 distinct antigens targeted by T cells. Disease cohort-specific analysis revealed that asthmatic patients recognize a broader epitope repertoire compared to rhinitic patients. The identification of T cell epitopes in mouse allergy reveals an immuodominant set of peptides that can be exploited for the detection of mouse-specific T cells *ex vivo*, revealing T cell phenotypes associated with different degrees of disease severity.

## Introduction

Mouse (MO) allergies are of growing importance in children and adults alike as they are potent sensitizers (1) and MO allergies are prevalent in the United States, especially in inner city populations (2, 3). A study of children in American inner cities reported that 18% have positive mouse skin test responses (1). Similarly, prevalence of mouse sensitization of 10–26% have been reported (4, 5) in cohorts of animal-care workers, exposed to MO allergens because of occupational duties. The clinical relevance of MO allergies is underlined by several studies indicating that MO-sensitization is a strong correlate of asthma development (6, 7). MO-specific IgE is associated with early wheeze and atopy in inner-city birth cohorts. The odds ratio for onset of wheezing by age 3 is 4.6 for MO-sensitized children and remarkably rises to 9.7 in children cosensitized to MO and cockroach (6), another pest-related allergy commonly found in inner cities. Furthermore, high IgE titers to MO and to German cockroach (CR) have also been associated with atopic dermatitis (6).

Despite their clinical and epidemiological importance, little is known about MO allergens at the molecular level. Studies on occupational allergies to small rodents and their molecular triggers date back several decades (8, 9). Sources of MO allergens include epithelium (10), urine (11), serum (12) saliva (13), hair and dander. Urine is the most potent source of MO allergens, since rodents have permanent proteinuria and are behaviorally prone to spray urine on their surroundings (especially males which have higher protein concentrations in urine). When urine dries up, proteins associate with airborne dust particles and can be inhaled, leading to sensitization.

Today, only one mouse allergen is listed in the IUIS database (14), namely Mus m 1, a major urinary protein (MUP) that belongs to the lipocalin superfamily (15). MUPs are encoded by a multigene family (*Mup* genes) and 8–14 MUPs are typically detected in a single adult mouse (16). The lipocalin superfamily includes several well-conserved mammalian allergens, including the major rat allergen Rat n 1, dog allergens Can f 1 and 2, horse allergen Equ c 1, cockroach allergen Bla g 4, and others (15).

Immunological studies of the molecular targets recognized by MO allergen-specific T cells are virtually non-existent. Over a decade ago, Jeal et al. performed a comprehensive T cell epitope mapping of the major rat allergen Rat n 1 (17), and epitopes have also been defined for Bla g 4 (18). In contrast, a query in the immune epitope database (IEDB) (19), a free resource that curates published human T cell epitopes for allergies and other diseases, only returned a single T cell epitope for mouse allergy published by Ferrari et al. (20). Therefore, we sought to fill this knowledge gap by identifying T cell epitopes recognized by MO-allergic individuals.

Moreover, it is currently unclear whether Mus m 1 is the only relevant MO allergen, or if other proteins are also of importance especially when T cell responses are considered. Indeed, mouse serum albumin has also been reported to have allergenic potential (12), though it is not officially listed in the IUIS. To address the question of whether, in addition to Mus m 1, other relevant T cell targets can be defined, we studied Mus m 1 isoforms/homologs, mouse homologs of mammalian allergens, and performed a broad immunoproteomic analysis of urine and epithelial extracts.

## Materials and Methods

### Study Population and PBMC Isolation

A cohort of 22 MO-sensitized patients and 10 MO-non-allergic, but MO-exposed patients, as defined by mouse-specific IgE titers of >0.35 kU_A_/L was studied. Patients were recruited from San Diego, CA, and New York City, NY, following Institutional Review Board approval (IRB protocols: VD-112-0217, GCO 13-0691). All patients enrolled in this study provided written consent. Clinical information is summarized in Table 1. The cohort was 59% female, with an age range of 23–61 years. IgE-titers were determined from plasma using the ImmunoCAP (Thermo Fischer, Uppsala, Sweden). PBMCs were isolated from whole blood by density gradient centrifugation according to manufacturers’ instructions (Ficoll-Hypaque, Amersham Biosciences, Uppsala, Sweden).

**Table 1:** Clinical and demographic data for all donor cohorts.

Donor	Birth year	Gender	Clinical status	Mouse IgE (kU_A_/L)	*In vitro* stimulus
1011	1979	M	Asthma	3.13	Urine
1209	1974	F	Asthma	3.09	Urine
1277	1965	M	Asthma	5.24	Urine
1284	1984	F	Asthma	2.71	Urine
1368	1994	M	Asthma	14.80	Epithelium
1424	1988	F	Asthma	57.60	Urine
1425	1979	F	Asthma	4.89	Urine
1435	1978	M	Asthma	9.01	Urine
1437	1980	F	Asthma	13.40	Urine
1440	1989	M	Asthma	2.14	Epithelium
1441	1971	M	Asthma	2.93	Urine
1460	1984	M	Rhinitis	2.49	Urine
1463	1993	M	Asthma	4.90	Urine
1600	1992	F	Rhinitis	1.02	Epithelium
1704	1985	F	Rhinitis	2.02	Urine
1726	1986	F	Asthma	2.68	Epithelium
2017	1994	M	ND	2.21	Urine
2397	1956	F	Asthma	1.08	Epith
2414	1979	F	Rhinitis	4.86	Epithelium
2423	1956	F	Rhinitis	0.71	Epithelium
2424	1980	F	Rhinitis	1.29	Epithelium
2489	1990	F	Rhinitis	11.00	Urine
1774	1985	F	Non-allergic	<0.1	n/a
2015	1987	M	Non-allergic	<0.1	n/a
2458	1982	F	Non-allergic	<0.1	n/a
2491	1992	M	Non-allergic	<0.1	n/a
2500	1981	F	Non-allergic	<0.1	n/a
2501	1992	M	Non-allergic	<0.1	n/a
2503	1979	F	Non-allergic	<0.1	n/a
2544	1991	F	Non-allergic	<0.1	n/a
2547	1992	F	Non-allergic	<0.1	n/a
2555	1989	M	Non-allergic	<0.1	n/a

### Selection of Peptides from Known Mouse and Other Mammalian Allergens

Sequences of two isoforms of the mouse allergen, Mus m 1 [Mus m 1.0101 and Mus m 1.0102, known as Mup 6 and Mup 2, respectively] (14) were collected from UniProt. The two sequences were aligned using MEGA software tool (21). 15mer peptides overlapping by 10 amino acids were generated to get the full coverage of both sequences. This peptide set included 34 peptides (peptide set #1, Figure S1 in Supplementary Material).

An additional set of twenty mouse urinary proteins was collected from GenBank and aligned with the Mup 6 and Mup 2 proteins. 15mer peptides overlapping by 10 amino acids were generated and a total of 172 additional peptides not included in the set of 34 peptides above were selected from this alignment. These 172 peptides were further screened for their predicted binding affinity as described in Paul et al. (22). A set of 48 additional Mus m 1-isoform -derived peptides with high binding affinity was selected (peptide set #2, Figure S1 in Supplementary Material).

The WHO/IUIS database (14) was screened for major mammalian allergens, and their sequences were blasted against the mouse genome (NCBI Protein Database). Thirty-one murine protein sequences homologous to known allergens from mammals were collected from GenBank using NCBI BLAST. A total of 244 peptides from 20 different proteins (Table S1 in Supplementary Material) were selected based on predicted binding affinity (22) and redundancy elimination (peptide set #3, Figure S1 in Supplementary Material). In addition, we selected the 244 corresponding mammalian peptides homologous to these murine peptides, to be used to evaluate specificity of responses (peptide set #4, Figure S1 in Supplementary Material). Some of the mouse sequences did not have corresponding regions in the mammalian allergen sequences because of sequence divergence and therefore the most identical peptides were selected.

### Selection of Peptides from Immunoproteomic Analysis

Methods for performing a 2-D immunoblot analysis to determine IgE and IgG reactivity have previously been described for cockroach (18) and Timothy grass (23) allergens. Briefly, urine and epithelia MO extracts (300 µg) were run on separate 2-D gels [3–10 pH range, 12% 138 (vol/vol) acrylamide] at Applied Biomics (Hayward, CA, USA). Subsequently, gels were blotted and the 2-D immunoblots were incubated with pooled plasma (diluted 1:50) from nine MO allergic donors (mouse IgE titers ranging from 4.9–56.7 kU/L) recruited in San Diego. Next, blots were incubated with goat antihuman IgE and mouse anti-human IgG (Sigma-Aldrich, Carlsbad, CA, USA), and MO donor antibody reactivity visualized using Cy2-conjugated donkey anti-goat IgG and Cy5-conjugated donkey anti-mouse IgG antibodies (Biotium, Fremont, CA, USA). We then determined the antibody reactivity of each spot by visual inspection of the 2-D immunoblot images. In total, 106 and 32 IgE and/or IgG-reactive protein spots for epithelial and urine extract were selected, respectively. Spots were cut out of gels run in parallel to the gels that were blotted. The cut out spots were analyzed by mass spectrometry. Proteins were identified by comparing the MS/MS spectral data to the mouse transcriptome using Mascot software (Matrix Science, Boston, MA, USA). The mass-spectrometry studies identified a total of 25 sequences from epithelial and 9 from urine extract. After redundancy elimination, a total of 23 proteins in the mouse epithelial extract gel not already covered by known allergen sequences described above were broken down to 1,356 peptides of 15mers size overlapping by 10 amino acids. A total of 307 peptides were selected based on the predicted binding affinity as described (22). Likewise, we observed nine proteins in the mouse urine gel of which four were already covered by peptides from the previous peptide sets. The remaining five proteins were again broken down to 15mers overlapping by 10 amino acids and a total of 85 predicted HLA class II binder peptides were thus selected from these proteins.

### Urine and Epithelia MO Extracts

Mouse epithelial extract was purchased from Greer (Lenoir, NC, USA). Mouse urine (mixed gender pooled, unfiltered) was purchased from CliniScinces (Nanterre, France). Low molecular components (<3 kDa) were removed by filtration centrifugation using Amicon Ultracel tubes (Merck Millipore, Darmstadt, Germany). The high-molecular-weight fraction (>3 kDa) was washed six times with PBS, each time followed by repeated centrifugation in Amicon Ultracel tubes with a cutoff of 3 kDa. The resulting urine extract was lyophilized and subsequently resuspended in PBS at 20 mg/ml (confirmed by BCA assay).

### HLA Typing and Inferred Restrictions

HLA typing for Class I (HLA-A; HLA-B; HLA-C) and Class II (HLA-DQA1; HLA-DQB1, HLA-DRB1,3,4,5; HLA-DPB1) was performed by an ASHI-accredited (American society for histocompatibility and immunogenetics) laboratory at Murdoch University (Western Australia) as previously described (24). Potential HLA-epitope restriction odds ratios and relative frequencies were calculated using the RATE program (25). To further filter identified inferred restrictions, HLA class II binding predictions were performed for peptide restrictions inferred by the RATE program, as recommended by the IEDB (19).

### Peptide Synthesis

Peptides were purchased from A and A (San Diego, CA, USA) as crude material on a small (1 mg) scale. Individual peptides were resuspended in DMSO at a final concentration of 40 mg/ml.

### Stimulation and Expansion of MO-Specific T Cells with Urine or Epithelia MO Extracts

For *in vitro* expansion of mouse-specific T cells, PBMCs of MO-sensitized individuals were stimulated with either epithelial (60 µg/ml) or urine extracts (3 µg/ml). Stimulation concentrations to induce optimal T cell responses were previously determined by titration (data not shown). Cells were cultured in RPMI 1640 supplemented with 5% human AB serum in 24-well plates (BD Bioscience, San Diego, CA, USA) at a density of 2 × 10^6^/ml and incubated at 37°C. IL-2 was added every 3 days after initial stimulation. Cells were harvested on day 14 and screened for IFNγ and IL-5-production by ELISPOT.

### Dual ELISPOT Assays

The production of IFNγ and IL-5 from cultured PBMCs in response to antigenic stimulation was assessed by dual ELISPOT assays as described previously (26). Cells (1 × 10^5^ cells/well) were stimulated with either peptide pools (5 µg/ml), individual peptides (10 µg/ml), or MO extracts (2 µg/ml each), PHA (10 µg/ml), or medium containing 0.25% DMSO (% of DMSO in the pools/peptides) as a control. Spot forming cells (SFC) were counted by computer assisted image analysis (KS-ELISPOT reader, Zeiss, Munich, Germany). T cell responses were background-subtracted and expressed per 10^6^ cells. Criteria for positivity were ≥ 20 SFCs per 10^6^ PBMCs, *p* < 0.05, and a stimulation index ≥ 2. Any responses that did not meet these criteria were set to 20 SFC, which is considered the sensitivity threshold for this assay. Positive pools (≥100 SFC) were deconvoluted to identify the individual epitopes inducing the response.

### Antigen-Reactive T Cell Enrichment (ARTE) Assay

*Ex vivo* T cell responses were measured based on T cell activation and cytokine production as previously described by Bacher et al. (27). Briefly, PBMC were thawed and rested overnight, plated at 10 × 10^6^ cells per well in a six-well plate. The next morning, cells were stimulated with urine (3 µg/ml), epithelial extract (60 µg/ml), peptide megapool (2 µg/ml), phorbol myristate acetate and Ionomycin (Io) (positive control), or medium alone (negative control) in the presence of 1 µg/ml CD40 (Miltenyi Biotech, Auburn, CA, USA). Cells were incubated for 6 h, adding Brefeldin A (1 µg/ml) for the last 3 h. After the incubation, cells were labeled with antiCD154-Biotin, anti-CD4 APC ef780, CD3 AF700, anti-CRTH2 Ax647, CD8/CD14/CD19 V500 and live/dead fixable viability dye (Life technologies, San Diego, CA, USA) followed by anti-Biotin MicroBeads (Miltenyi Biotech, Auburn, CA, USA). After staining and washing, CD154 + cells were magnetically enriched using MS columns (Miltenyi Biotech, Auburn, CA, USA). Fixation, permeabilization and intracellular staining was performed on the column using the Inside stain kit (Miltenyi Biotech, Auburn, CA, USA), anti-IL-4 BV421, IFNγ PerCPCy.5.5, anti-IL-10 AF488, anti-IL-17 PECy7, and CD154 PE (BD, San Diego, CA, USA). Finally, cells were elusted off the column and analyzed by flow cytometry using a BD LSR II flow cytometer and data were analyzed using FlowJo software (TreeStar, Ashland, OR, USA). All data acquisition was performed blinded.

## Results

### Mus m 1-Derived Immunodominant T Cell Epitopes

**Figure 1:**
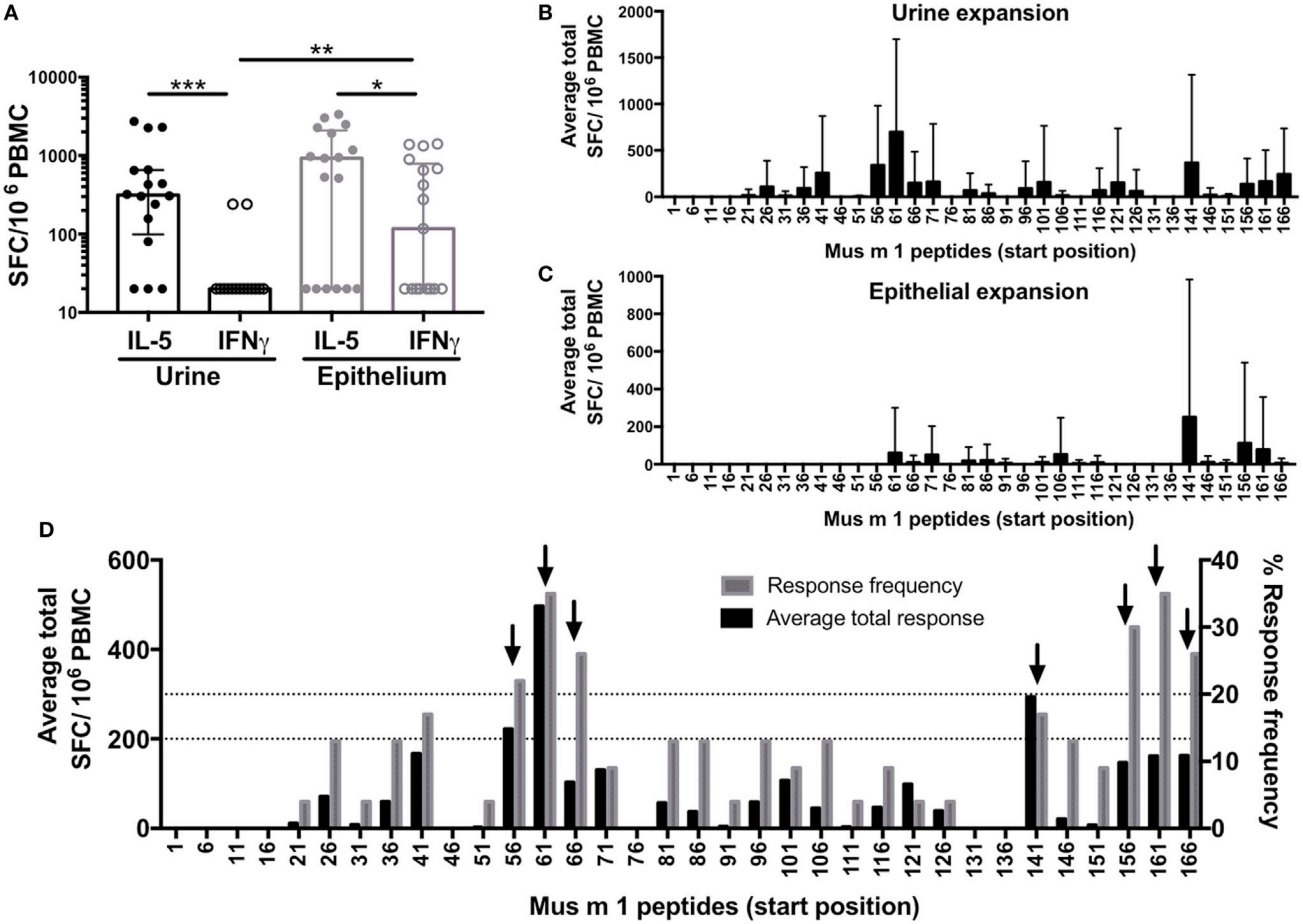
Allergic T cell responses to mouse urine and epithelial extract and the major allergen Mus m 1. Cytokine production was measured by ELISPOT following *in vitro* restimulation with **(A)** urine or epithelial extract; or overlapping 15-mer peptides spanning Mus m 1 after *in vitro* culture with **(B)** urine or **(C)** epithelial extract. Error bars indicate SDs. **(D)** Data from Mus m 1-derived peptide responses after urine and epithelial expansion were combined to identify immunodominant peptides [frequency ≥ 20%; magnitude ≥ 200 spot-forming cells (SFC)], indicated by black arrows. Responses below detection threshold were set to 20 SFC. Statistical analysis was performed by Wilcoxon signed rank test (paired), one-tailed comparison for IL-5 vs. IFNγ, and Mann–Whitney test (unpaired), two-tailed comparison for IFNγ vs. IFNγ. *p < 0.05, **p < 0.01, and ***p < 0.001.

To compare potencies of epithelial and urine extracts to expand MO-specific T cells from allergic donors *in vitro*, we measured extract-specific IL-5 and IFNγ production by ELISPOT in urine (*n* = 15) or epithelial (*n* = 17) expanded cells following 24 h restimulation with the extract used for expansion. Overall, both extracts expanded T cells with similar efficiency (Figure 1A). While both extract responses were dominated by IL-5, this polarization was most pronounced for urine extract, while significantly higher IFNγ production was observed in response to the epithelial extract.

To define Mus m 1 T cell epitopes recognized in MO allergic individuals, PBMC from allergic donors were expanded with either urine or epithelial extracts, and cytokine responses (IL-5 and IFNγ) were determined following 24 h restimulation using overlapping peptides spanning the entire Mus m 1 sequence. Not surprisingly, the urine extract was more efficient in expanding cells responding to restimulation with Mus m 1 as compared to the epithelial extract. However, the epitopes recognized after short-term restimulation following epithelial extract only comprise a fraction of those recognized after urine expansion (Figures 1B–C) and reactivity after epithelial expansion was lower overall compared to urine. The combined data from all 22 allergic donors tested (8 epithelial expanded, 14 urine expanded) revealed seven dominant Mus m 1 epitopes (defined as those with magnitude of ≥200 SFC and/or frequency ≥ 20%) (Figure 1D), corresponding to three main T cell-reactive regions.

### Conservation of Mus m 1 within Mouse and Other Rodents Is a Determinant of Immunodominance

Mouse Urinary Proteins (MUPs) include several Mus m 1 isoforms, and other highly related proteins. A comprehensive panel of 48 peptides derived from various MUPs with a high predicted binding affinity to MHC molecules (22) was screened by ELISPOT following urine (*n* = 14) or epithelial (*n* = 8) extract expansion. In ten instances, responses were detected against both the Mus m 1 peptide and a homologous isoform version (Table S2 in Supplementary Material). Seven of those ten instances corresponded to the seven immunodominant peptides identified above (Figure 1D). This suggests that sequence conservation in different allergens is a determinant for immunodominance, as previously reported in pollens and other systems (28).

**Figure 2:**
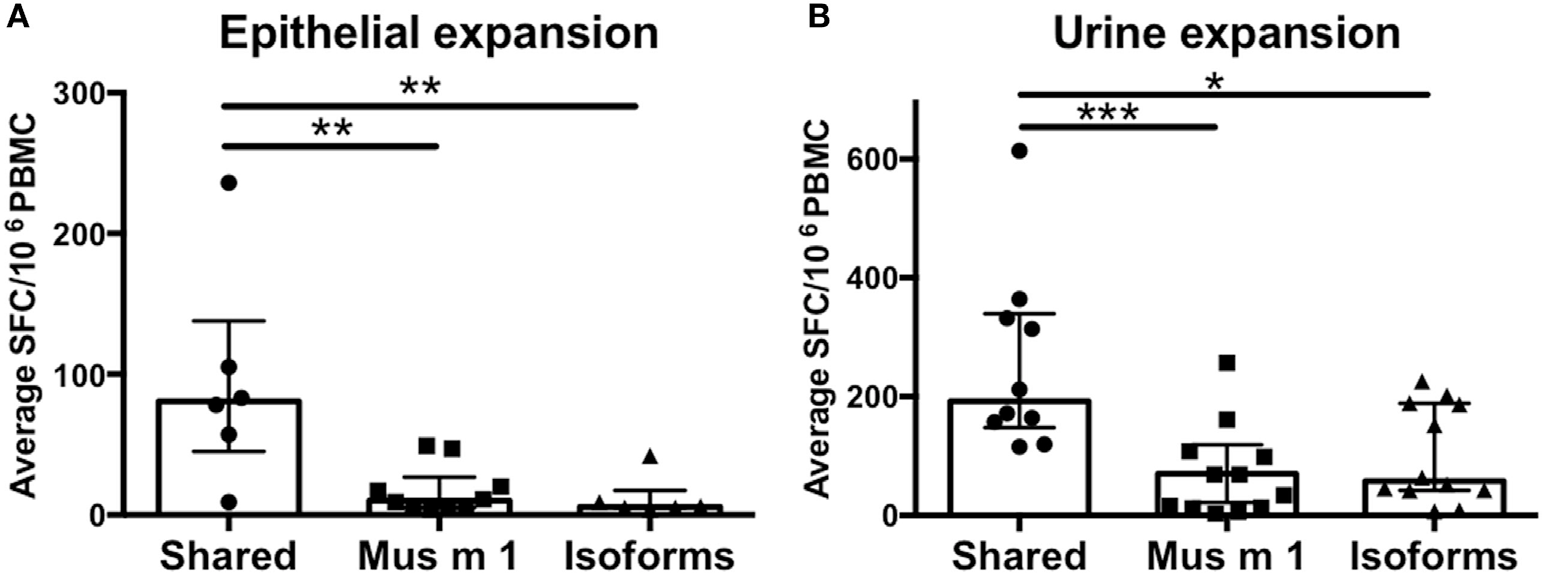
T cell reactivity against conserved vs. non-conserved major urinary protein (MUP) peptides. Cytokine production (IL-5 + IFNγ) was measured as spot forming cells (SFC) by ELISPOT in response to Mus m 1-unique, other MUP isoforms-unique, or conserved (shared) peptides after expansion with **(A)** epithelial extract or **(B)** mouse urine. Statistical analysis was performed by Mann–Whitney test, one-tailed. *p < 0.05, **p < 0.01, and ***p < 0.001.

Next, the data derived from screening of all MUPs as a whole, including Mus m 1, was further analyzed (Figures 2A,B). Peptides for which T cell reactivity was observed against both Mus m 1 and the homologous isoform peptide were defined as “shared.” Peptides with T cell reactivity only against either the Mus m 1 or the isoform peptide were defined as “unique.” We found that shared peptides elicit significantly higher T cell responses compared to unique peptides. Out of 42 T cell epitopes, 29 peptides were shared, and 13 peptides were unique, compared to the total peptide set tested which contained 41 shared peptides and 41 unique peptides (one-sided Fisher’s exact test *p* = 0.03). These results confirmed that isoform conservation is a major determinant of immunodominance within MUPs.

A previous study reported a set of 19 T cell epitopes (17) from the major rat allergen, Rat n 1, also a MUP with significant homology to Mus m1. Here, the IEDB epitope clustering tool (Dhanda et al., submitted) was used to identify T cell-reactive peptides conserved between Mus m 1 and Rat n 1 (Table S3 in Supplementary Material). Eight of the 20 Mus m 1 epitopes clustered with Rat n 1 epitopes at the 70% homology threshold. Six of those eight are among the top nine epitopes in terms of strength of reactivity. This association is statistically significant (*p* =0.023 by Fisher’s exact test, one-tailed). This finding supports the notion that peptides conserved in other rodent species elicit a more dominant immune response, possibly due to increased/repeated exposures by the various homologous allergen species.

### T Cell Reactivity against MO Orthologs of Allergens from Other Mammals

Several allergens have been described in other mammalian species, such as horses, cattle, rabbits, guinea pigs, hamsters, cats and dogs. In most cases, these proteins have mouse orthologs. Here we hypothesized that at least some of these mouse orthologs could also have allergenic potency. To address this issue, we tested 244 predicted HLA class II binding peptides from orthologs to 20 known mammalian allergens derived from 7 different animals (Table S2 in Supplementary Material). To determine whether T cell reactivity detected against the mouse sequences was due to cross-reactivity between the mouse and homologous mammalian sequence, we also tested in parallel, for each peptide, the corresponding peptide from the originally described mammalian allergen.

**Figure 3:**
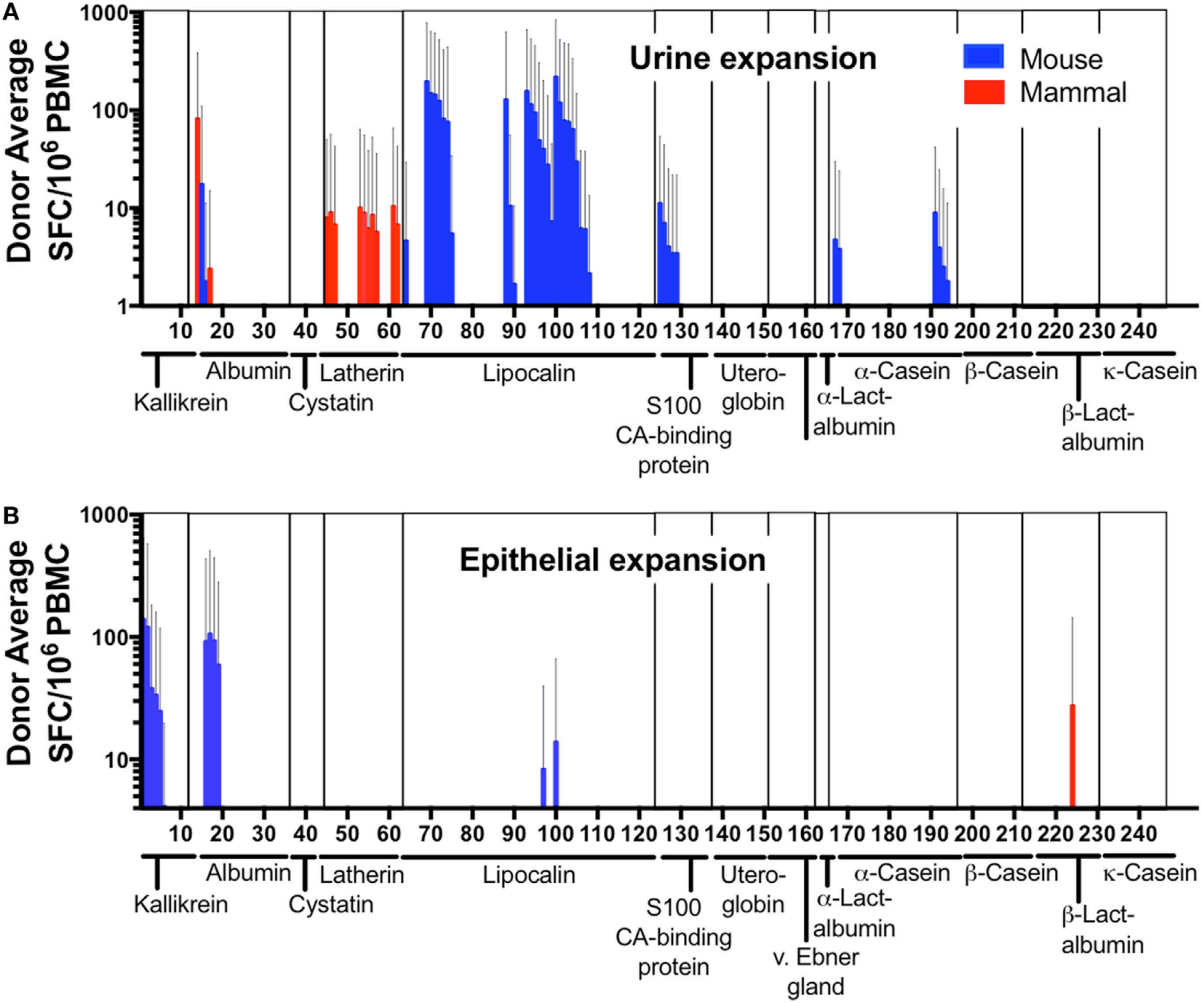
T cell reactivity against mammalian allergens and their murine homologs. Cytokine production (IL-5 + IFNγ) was measured as spot forming cells by ELISPOT in response to mammalian-allergen-derived peptides and their homologous murine counterpart after expansion with **(A)** mouse urine or **(B)** epithelial extract. Allergen families are indicated along the *x*-axis. Error bars indicate SDs.

Out of a total of 244 peptide pairs tested, responses were detected against 61 MO-derived peptides from 12/20 mouse orthologs of known mammalian allergens (Figures 3A,B). A strong bias was observed for urine extract to expand lipocalin-specific T cells (Figure 3A), while stimulation with epithelial extract expanded mostly albumin and kallikrein-specific T cells (Figure 3B). Actual cross-reactive recognition (reactivity against both the mouse peptide and its mammalian homolog) was only detected in a single case, namely Cav p 6, the albumin from guinea pig and its mouse homolog (Figure 3A), suggesting that the majority of these proteins are bona-fide mouse T cell antigens. Furthermore, some T cell responses against mammalian peptides in absence of reactivity to the mouse homolog were also observed, targeting 13 peptides from 3 allergens (Bos d 5, Cav p 4 Equ c 4), suggesting that in these cases, donors may be polysensitized against MO and other animal allergens. In conclusion, these data identified several novel mouse antigens targeted by T cells, based on the fact that their orthologs in other mammalian species were known allergens.

### A Global View of Immunodominance in MO Responses

**Figure 4:**
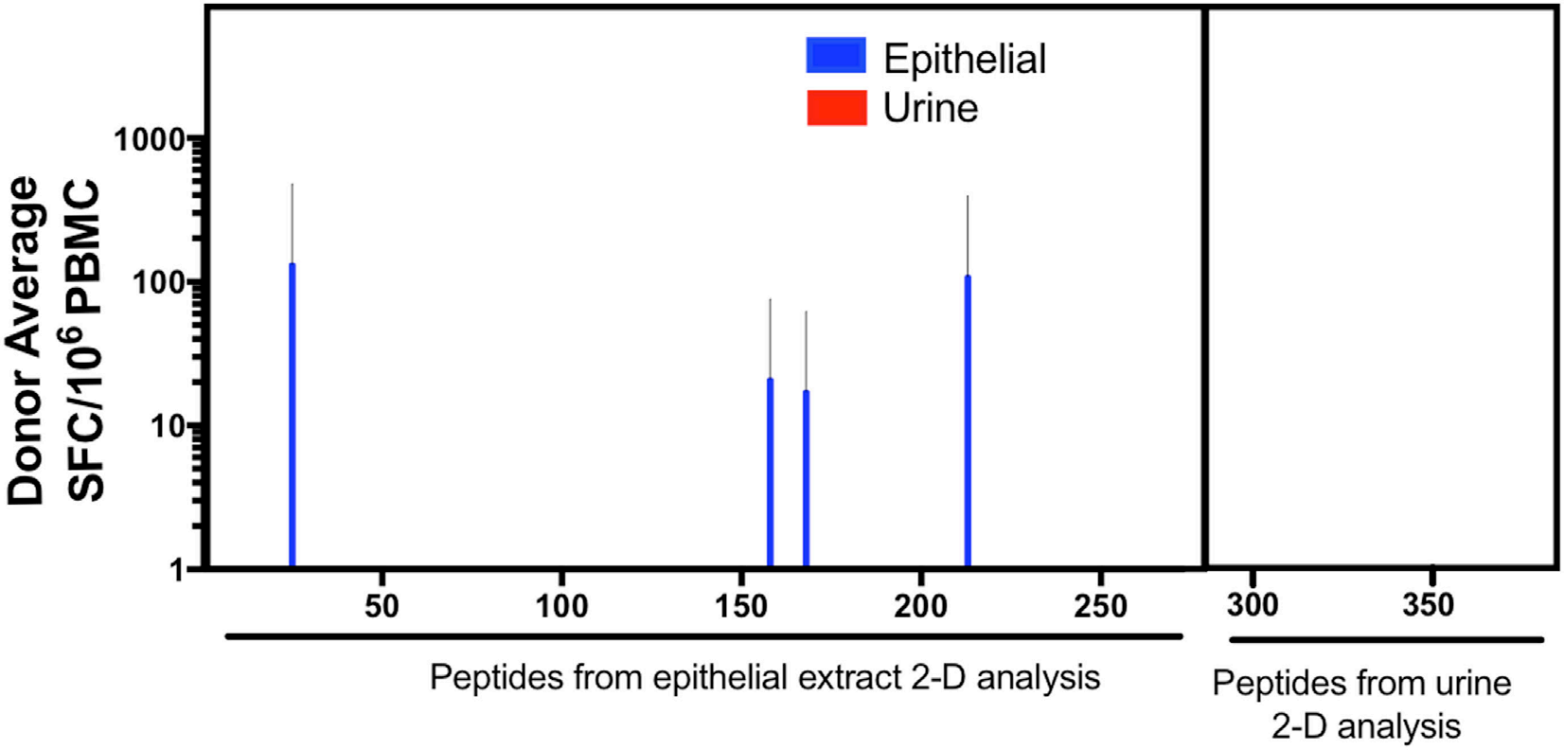
T cell responses against previously undescribed IgG and IgE-reactive mouse proteins. Cytokine production (IL-5 + IFNγ) was measured as spot forming cells (SFC), by ELISPOT in response to predicted peptides derived from immunoproteomic analysis of mouse urine and epithelium. Error bars indicate SDs.

To broadly define additional potential murine antigens, we followed a previously described immunoproteomic approach (18, 23). As described in the methods, peptides predicted from proteins identified based on their IgE and IgG-reactivity profile from 2D immunoblots were screened for T cell reactivity after urine and epithelial extract expansion. Epithelial extract stimulation expanded T cells reactive to peptides from 4 out of 23 novel proteins identified from 2D immunoblot analysis of epithelial extract (Figure 4). These proteins included Alpha-amylase [a known allergen cockroach (29, 30) among others (31)] and two fragments of the murine Ig kappa chain. No reactivity was observed against any further novel proteins obtained from urine 2D immunoblot analysis, regardless of culture stimulus. Thus, the immunoproteomic analysis revealed relatively few additional novel T cell targets.

Based on this comprehensive set of data, we analyzed the patterns of T cell immunodominance in mouse allergy. After elimination of redundant peptides with ≥70% identity using the clustering tool (Dhanda et al., submitted), a total of 106 epitopes were recognized (see complete list in Table S4A in Supplementary Material). The top 26 epitopes account for 75% of the response, and the top 40 epitopes make up 90% of the total response. At the antigen level, 79% of total response is accounted for by Lipocalin-related proteins (Table S4B in Supplementary Material) with Mus m 1 alone accounting for 27% of the total response, making it the most dominantly recognized T cell antigen. The top 7 antigens (out of 35 in total) account for ~75% of the response, and of those, 5 are Lipocalins. Similarly, out of 12 antigens accounting for 90% of the response, 9 are Lipocalins.

### HLA Restriction Predictions Using RATE

The issue of which HLA restriction is associated with a given epitope is of considerable interest. HLA types of all donors included in this study were determined by HLA typing (Table S5 in Supplementary Material). A genetic inference method, named restrictor analysis tool for epitopes (RATE) (25), was used to infer HLA restriction of epitopes from T cell response data in HLA typed subjects. While the inferred restrictions here are based on a limited set of donors (*n* = 22), nevertheless it appears that all four HLA class II loci (DRB1; DRB3, DQ, and DP) appear to be restricting responses (Table 2). These patterns of inferred HLA restriction should be interpreted with caution, since the small number of donors analyzed limits the power of HLA restriction assignments based on genetic inference. We expect that as data relating to more donors becomes available, more genetic associations, relating to more allelic variants will become apparent, especially as it relates to promiscuous restriction to several HLA class II molecules.

**Table 2:** Inferred HLA allele restriction analysis performed using the RATE analysis tool.

Antigen	Peptide seq	Allele#	Allele	A+R+	A−R+	A+R−	A−R−	No. of donors	Relative freq	Odds ratio	*p*-Value	%ile binding
Mus m 1	FRLFLEQIHVLENSL	32	DQB1*05:01	4	1	2	15	22	2.9	30	0.0093	5.11
Mus m 1	EPDLSSDIKERFAQL	42	DRB1*03:01	3	1	1	17	22	4.1	51	0.0100	0.18
Mus m 1	EPDLSSDIKERFAKL	42	DRB1*03:01	3	0	1	18	22	5.5	Inf	0.0026	0.17
MUP 14	EEASSTGRNFNVEKINGEWHTII	61	DRB3*01:01	3	0	2	17	22	4.4	Inf	0.0065	16.48
MUP 13	GLYGREPDLSSDIKERFA	42	DRB1*03:01	3	0	1	18	22	5.5	Inf	0.0026	0.59
MUP 11	GKYSVTYDGFNTFTI	8	DPB1*04:02	3	1	0	18	22	5.5	Inf	0.0026	17.35

*p < 0.05 is considered significant*.

*A, allele; R, responder; RF, relative frequency; OR, odds ratio*.

### Differences in Antigen-Recognition But Not Magnitude or Polarization Differentiate Asthmatic vs. Rhinitic T Cell Responses

**Figure 5:**
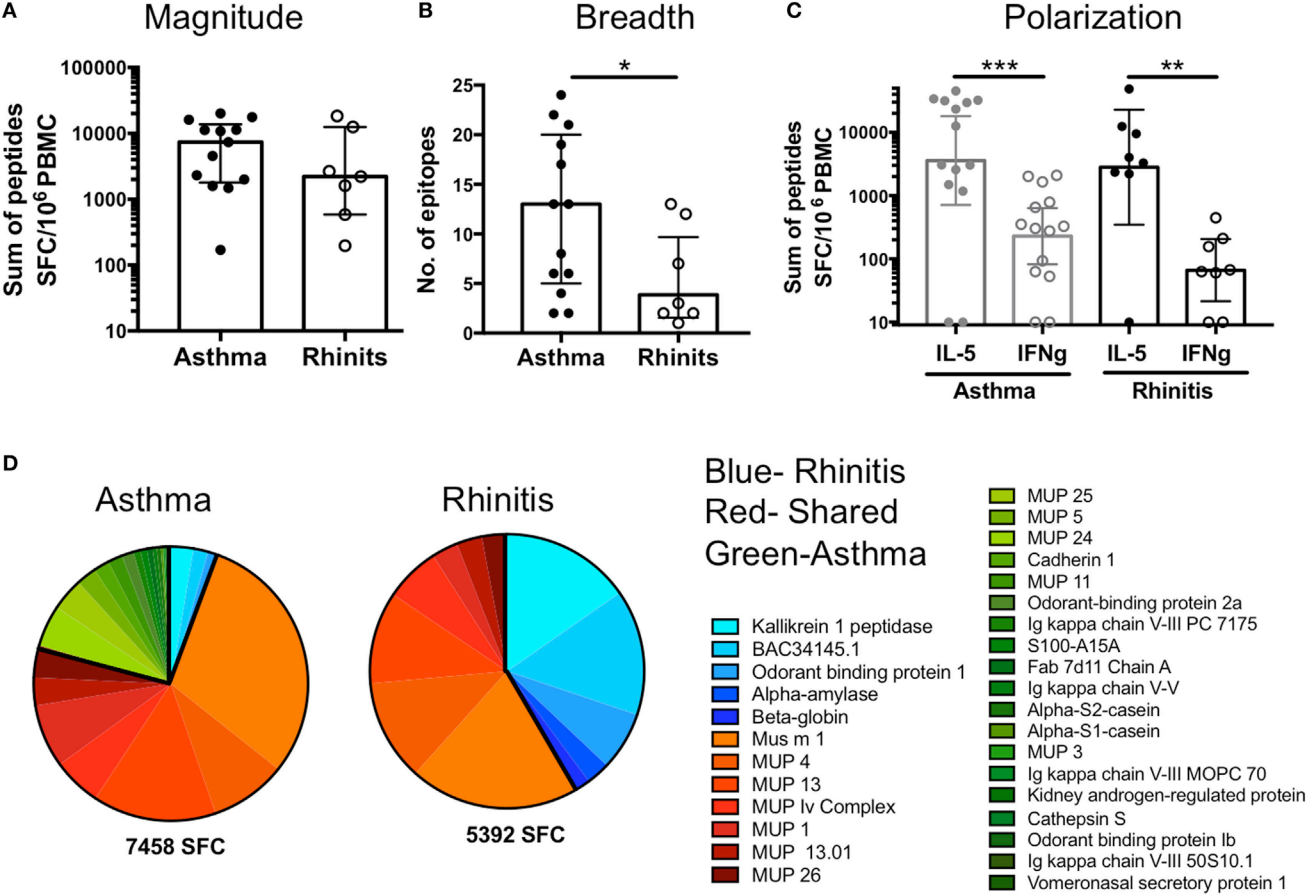
Differences in T cell reactivity in asthmatic versus rhinitic donors. **(A)** Response magnitude, **(B)** breadth of response, **(C)** cytokine polarization and **(D)** antigen immunodominance were assessed in asthmatic (*n* = 13) and rhinitic (*n* = 7) donors. Antigens predominantly recognized in asthmatic donors are shown in green, antigens recognized by both cohorts are shown in red and antigens predominantly recognized by rhinitic donors are shown in blue. Statistical analysis was performed by Mann–Whitney test, one-tailed. *p < 0.05, **p < 0.01, and ***p < 0.001.

In the German cockroach allergy system (18) different clinical phenotypes are associated with different T cell response magnitude and epitope specificity, and these differences can be used to discriminate asthmatic patients from non-asthmatic, allergic rhinitis patients. To determine if similar differences in T cell specificity are also observed in mouse allergy, we separately analyzed asthmatic (*n* = 13) and non-asthmatic, rhinitis donors (*n* = 7) (Table 1) (one donor was unresponsive to all dominant 106 epitopes, one donor was of unknown disease status). At the individual patient level, no difference in response magnitude against single epitopes was observed (Figure 5A). However, when comparing the breadth of epitope reactivity (number of epitopes recognized), asthmatic patients had an increased breadth of response compared to rhinitic patients (Figure 5B). Both asthmatic and rhinitc responses were strongly IL-5-polarized (Figure 5C), consistent with observations of whole extract T cell reactivity (Figure 1A). Asthmatic donor responses were more diverse compared to rhinitic donor responses (33 vs. 12 antigens, respectively; overlap of 10 antigens) (Figure 5D). Ten antigens elicited T cell reactivity in both cohorts, accounting for 73% of the total response in asthmatics and 58% in rhinitc patients (Figure 5D). The other antigens were rather selectively recognized in either asthmatic or rhinitic donors. Rhinitc donors exhibited a more focused response, with 42% of it targeting 5 antigens (Kallikrein, BAC34145, Odorant binding protein, Alpha-amylase and Beta-goblin) that made up less than 6% of the reactivity in the asthmatic cohort. In parallel we noted that 21% of the T cell response in asthmatics targeted antigens that were not recognized at all by rhinitic patients (Figure 5D).

### An Epitope Megapool as a Tool to Study Mouse-Specific T Cell Responses *Ex Vivo*

**Figure 6:**
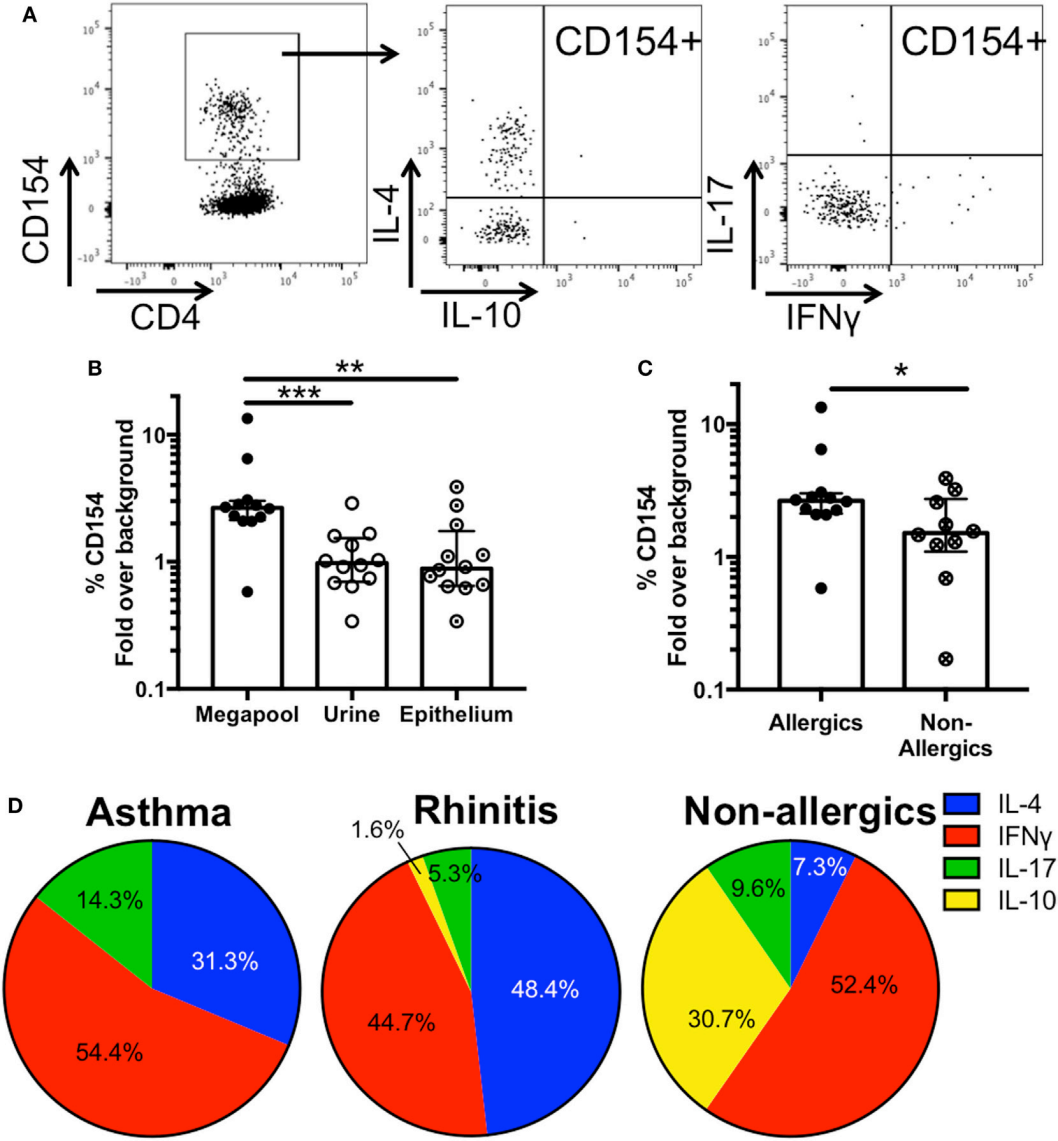
*Ex vivo* T cell activation and cytokine production in response to mouse antigen. **(A)** Representative FACS plots showing CD154+ cells and intracellular cytokine staining in activated cells after megapool stimulation **(B)** *Ex vivo* T cell activation in response to mouse epitope megapool or extract stimulation was assessed based on CD154 expression (*n* = 12). Wilcoxon test (one-tailed) was used for statistical analysis. **(C)** *Ex vivo* T cell activation in response to mouse epitope megapool in mouse allergic (*n* = 12) and non-allergic individuals (*n* = 10). Mann-Whitney test (one-tailed) was used for statistical analysis. **(D)** Patterns of cytokine production in antigen-specific (CD154+) T cells in asthmatic (*n* = 6), rhinitic (*n* = 6), and non-allergic (*n* = 10) donors. *p < 0.05, **p < 0.01, and ***p < 0.001.

Characterizing allergen-specific T cell responses *ex vivo* is challenging because the frequency of these cells in peripheral blood is often at the limit of detection. We previously demonstrated that allergen-specific T cells are detectable *ex vivo* using pools of dominant T cell epitopes (32) in the House Dust Mite system. Following the same approach, we created a mouse allergy epitope megapool, consisting of 106 dominant T cell epitopes identified herein, and assessed its ability to elicit T cell reactivity directly *ex vivo* in cells from 12 mouse allergic patients (6 rhinitic, 6 asthmatic), for whom we had sufficient cells left. Using the upregulation of the activation marker CD154 (CD40L) as a read-out for T cell reactivity, we used the previously published Antigen-Reactive T cell Enrichment assay (27) to detect mouse-specific T cells after short-term stimulation with epithelial extract, urine extract or the epitope megapool. In addition, intracellular cytokine staining was performed, amending the previous assessment of cytokine production from IL-5 and IFNγ to assessing production of IL-4, IFNγ, IL-17, and IL-10 (Figure 6A) to determine if different patterns of cytokine production are associated with asthmatics or rhinitic disease status. IL-5 was not measured *ex vivo*, as in our experience its detection by flow cytometry is far inferior compared to ELISPOT.

Stimulation with the megapool successfully elicited epitope-specific T cell activation > 2-fold above background (medium alone) in 11 out of 12 donors, whereas only 1/12 and 2/12 donors were reactive after stimulation with urine and epithelial extract, respectively (Figure 6B). These data suggest that stimulation with the top T cell epitopes greatly improves the detection of low-frequency mouse-specific T cells *ex vivo*, which are largely undetected after extract stimulation.

Analysis of the average cytokine production in CD154+ T cells revealed that responses were dominated by IFNγ and IL-4 in both asthmatic and rhinitic patients (Figure 6B; Table S6 in Supplementary Material). However, 14.3% of the total cytokine response in asthmatics was accounted for by IL-17, which was almost threefold lower (5.3%) in rhinitic patients. IL-10 made up less than 2% of the total cytokine production in rhinitic patients and was totally absent in asthmatics (Figure 6D). To assess whether the epitope megapool is preferentially recognized in allergic as opposed to non-allergic donors, cells from 10 MO non-allergic donors, who are exposed to mice every day due to occupation, were also assessed. *Ex vivo* T cell activation to the mouse megapool was significantly lower in allergics compared to non-allergics (*p* = 0.02) (Figure 6C). Furthermore, non-allergic individuals exhibited a very different cytokine profile, dominated by IFNγ and IL-10 and very little IL-4 and IL-17 (Figure 6D).

## Discussion

Despite its clinical importance, little is known about the human allergic immune response to mouse antigens, especially on the T cell level. To the best of our knowledge, this is the first study focused on mouse-specific T cell responses in mouse-sensitized asthmatic and rhinitic patients. We found that Mus m 1 (lypocalin) and serum albumin are strongly dominant antigens at the T cell level, with Mus1 being dominant in particular in urine extract and albumin more represented in the epithelial extract. A more in depth analysis revealed that sequence conservation across lypocalins played an important role in determining immunodominance. Our studies also revealed several minor T cell antigens, mostly identified through screening of orthologs of reported mammalian allergens and, in very few cases by immunoproteomic analysis of 2D immunoblots of mouse extracts. Interestingly, the T cell response specificity differed depending on clinical phenotype, with mouse-sensitized asthmatic and rhinitic patients recognizing only partially overlapping sets of antigens.

It has to be mentioned that the protein extracts and peptides were not subject to extensive purification, therefore some responses may have been affected by endotoxins present in the materials. However, not all extracts and peptides elicited responses in all cultures suggesting that non-specific reactivity did not influence our findings in a major way.

The use of epithelial extract in the diagnosis of mouse allergy has been a matter of debate in the field due to its low and variable amounts of Mus m 1, the only known major mouse allergen. For this reason, a previous study (33) investigated the diagnostic utility of mouse urine and compared it to the performance of commercial epithelial extract. They reported both extracts to be of comparative diagnostic performance, suggesting in addition to Mus m 1, other proteins such as albumin may also be important triggers for clinical symptoms to mice. This is in line with our finding that both extracts are comparable in the stimulation of MO-allergen-specific T cells.

We identified seven immunodominant epitopes from Mus m 1, the only known mouse allergen registered in the IUIS database (14). To the best of our knowledge, only one Mus m 1-derived T cell epitope had been reported to date (20), which largely overlaps with one of our seven dominant T cell epitopes (Mus m 1_141_). When we extended our T cell epitope mapping efforts beyond Mus m 1, investigating peptides from Mus m 1 isoforms, mammalian allergen orthologs and protein targets identified by immune-proteomic analysis of mouse urine and epithelial extract, we found that Mus m 1 alone accounted for 27% of the total T cell response, confirming the dominant role of this allergen in mouse allergy. Further extending this analysis to consider other members of the lipocalin superfamily (Mus m 1 isoforms and mammalian homologs) revealed that lipocalins accounted for 79%, while other T cell-reactive antigens, including serum albumin, only made up 21% of the response. These data suggest that the mouse allergic T cell response is very focused, potentially making it an attractive model system for single allergen or even peptide-based immunotherapy approaches. However, it is important to mention that this screen was limited to IL-5 and IFNγ production, therefore epitopes eliciting exclusively other cytokine responses such as IL-10 or IL-17 will not have been detected.

The impact of sequence conservation on immunodominance has been reported in many different systems, including grass pollen allergy (28), viral infection (34, 35) and tuberculosis (36). Mus m 1 is part of an allergen family that is highly conserved among several mammalian species. An analysis of the impact of sequence homology on Mus m 1-specific T cell reactivity revealed that peptides that are shared between Mus m 1 and other MUPs are significantly more T cell-reactive, suggesting that also in mouse allergy, sequence conservation ultimately boosts T cell responses, presumably due to increased frequency of exposure.

Screening orthologs of reported mammalian allergens revealed several additional T cell antigens, showing that a systematic ortholog approach can be used to screen for T cell targets in mammals. Conversely, the immunoproteomic approach, previously applied to cockroaches, pollens and house dust mites (18, 23, 37) yielded only few hits. The reasons for this are not clear but might be related to the immunodominance of lypocalins, as discussed above.

Sensitization to mouse is strongly associated with wheezing in children and asthma in adults, however, some mouse-allergic patients never develop any asthmatic symptoms. Here we found no difference in magnitude of responses, but rather we uncovered that the breadth of response, both at the antigen and epitope level, is significantly higher in asthmatics compared to rhinitis, which was focused on fewer but potentially more dominant antigens. Interestingly, the T cell response specificity in mouse-sensitized asthmatic and rhinitic patients only partially overlapped, as the cohorts shared recognition of 10 of the 35 T cell-reactive antigens. A similar difference in the antigens recognized by asthmatic versus rhinitis patients was noted in CR-allergic donors (18). The mechanism underlying these observations is not clear, but it might be related to cosensitization and cross-reactivity to other allergens in these cohorts.

Finally, we assessed whether the most dominant T cell epitopes could be used as a tool to facilitate the detection and phenotypic characterization of mouse-specific T cells directly *ex vivo*. Using the upregulation of the T cell activation marker CD154 as a read-out, we found that mouse-specific T cells could be detected in >90% of donors, whereas urine or epithelium extract stimulation only triggered CD154 expression in 8.3 and 16.6% of donors, respectively. Moreover, analysis of cytokine production in CD154+ T cells of asthmatic and rhinitic patients revealed that, while both cohorts predominantly express IFNγ and IL-4, asthmatics produce almost three times more IL-17 on average. This is consistent with studies suggesting a central role for IL-17 in asthma based on levels in sputum and tissue biopsies from asthmatic patients (38). Of note, *ex vivo* responses appear to be less dominated by Th2 cytokines compared to T cell reactivity after expansion measured by ELISPOT. The reason for this is not fully clear, however, it may be related to the fact that the different assays exhibit different sensitivity for IFNγ detection or that the 14-day culture environment favors Th2 proliferation.

Interestingly, comparison of *ex vivo* T cell activation in allergic and non-allergic donors revealed a significantly decreased reactivity in non-allergics, associated with a Th1/Tr1 dominated cytokine production profile.

Sensitization to mice is an important risk factor for asthma development, yet very little data is available on the immunological targets recognized by the human immune system. Our investigation of T cell epitopes recognized in mouse allergy allowed the creation of an epitope megapool, which enables detection and phenotypic characterization of mouse-specific T cells directly *ex vivo*. These data provide a good basis for future studies to improve mouse allergy diagnostics and increase our understanding of the immunopathology associated with MO-allergies.

## Ethics Statement

Patients were recruited from San Diego, CA, and New York City, NY. following Institutional Review Board approval (IRB protocols: VD-112-0217, GCO 13-0691). All patients enrolled in this study provided written consent.

## Author Contributions

LW and GB: experimental design and performance. SP: bioinformatical analyses. PB: clinical sample collection and preparation and scientific input. JS: peptide manufacture and scientific input. VS, BP, and AS: project conception, planning, experimental design, data analysis, and manuscript preparation.

## Conflict of Interest Statement

The authors declare that the research was conducted in the absence of any commercial or financial relationships that could be construed as a potential conflict of interest.
