# Supplementary material for: Allergen and Epitope Targets of Mouse-Specific T Cell Responses in Allergy and Asthma
Source: Front Immunol. 2018 Feb 13;9:235. doi: 10.3389/fimmu.2018.00235 (PMC5816932; doi:10.3389/fimmu.2018.00235)
Supplement: Supplementary file 1 [file data_sheet_1.docx]

**Supplemental information**

Online methods:

Peptide binding prediction: 7-allele method

A large number of proteins with potential allergenic potency were identified from other mammals, murine epithelial and urine extracts and Mus m 1 isoforms. It was not feasible to screen overlapping peptides from these various antigens with the amount of blood provided. Therefore, MHC class II binding was predicted from overlapping peptides generated from sets 2-6. The prediction was done using the 7-allele method described in detail previously([Paul et al., 2015](#_ENREF_1)). Briefly, this method was design to predict the most dominant epitopes, at the population level, and is not focused on any particular HLA type. To derive this “general and agnostic predictor” we used human immunogenicity data associated with sets of 15-mers overlapping by 10, spanning over 30 different allergens and bacterial antigens and HLA class II binding prediction tools, we optimized a strategy to predict the top epitopes recognized by human populations. The most effective strategy was to select peptides based on predicted median binding percentiles for a set of seven HLA class II allelic motifs, representative of the more common general HLA -peptide binding modes.

References:

Paul, S., Lindestam Arlehamn, C.S., Scriba, T.J., Dillon, M.B., Oseroff, C., Hinz, D., Mckinney, D.M., Carrasco Pro, S., Sidney, J., Peters, B., and Sette, A. (2015). Development and validation of a broad scheme for prediction of HLA class II restricted T cell epitopes. *J Immunol Methods* 422**,** 28-34.


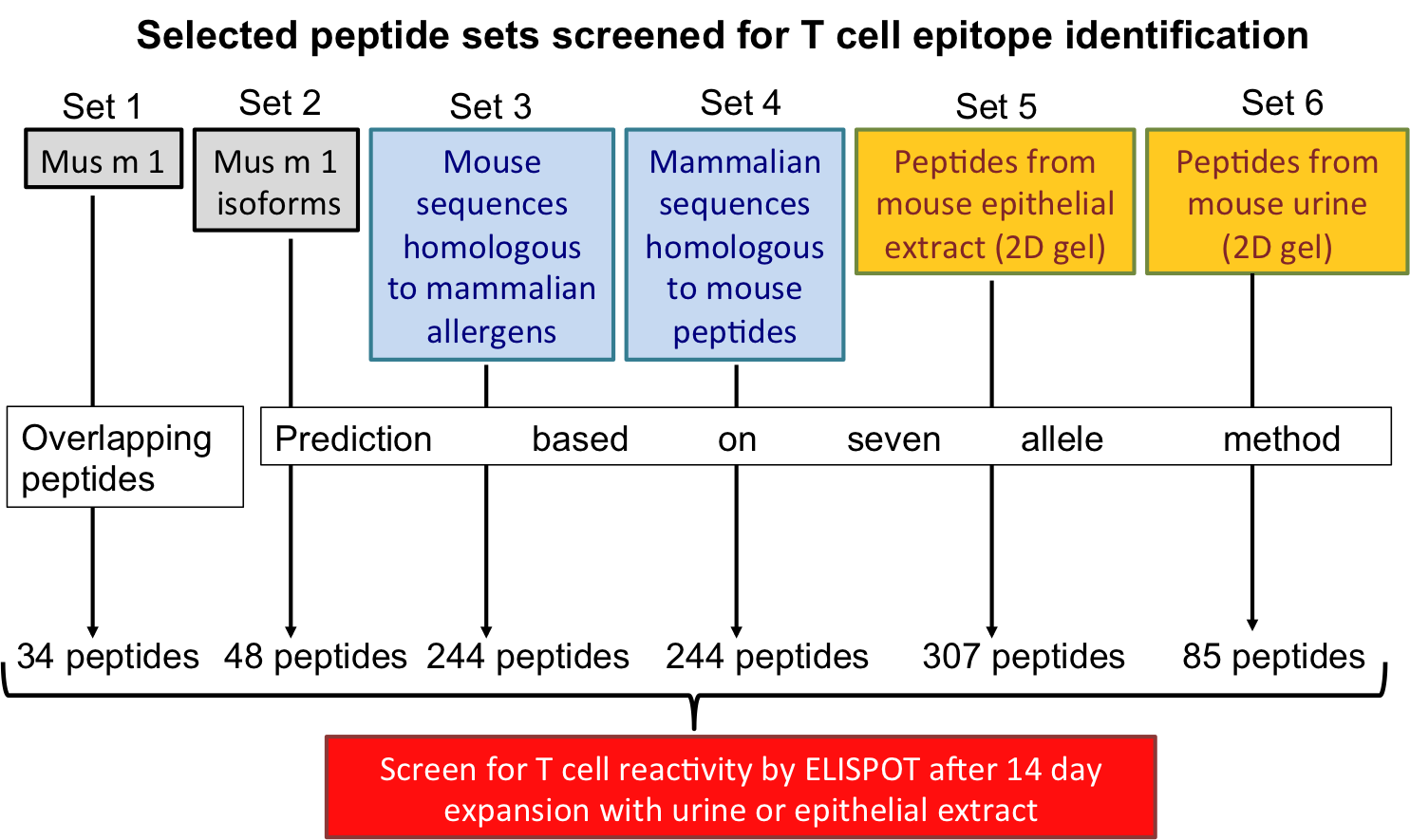


Supplemental figure 1. A graphical summary of the peptide sets tested.

Supplemental table 1. A summary of mammalian allergens for which murine orthologs were screened.

|  |  |  |  | **Homology to mouse** | |
| --- | --- | --- | --- | --- | --- |
| **GI number** | **Allergen** | **Mammal** | **Protein family** | **% Identity** | **% Positives** |
| 11993600 | Ory c 3 | Rabbit | Lipocalin | 40.2 | 68.5 |
| 126514234 | Equ c 4 | Horse | Latherin | 28.6 | 55.0 |
| 13124669 | Mes a 1 | Golden Hamster | Lipocalin | 60.1 | 75.7 |
| 1575778 | Equ c 1 | Horse | Lipocalin | 49.4 | 71.4 |
| 163283 | Bos d 4 | Cattle | α-Lactalbumin | 62.7 | 73.9 |
| 163823 | Fel d 1.0101 | Cat | Uteroglobin (chain 1) | 36.0 | 55.0 |
| 163825 | Fel d 1.0102 | Cat | Uteroglobin (chain 1) | 58.1 | 73.1 |
| 2598974 | Can f 1 | Dog | Lipocalin | 42.4 | 66.3 |
| 27806963 | Bos d 10 | Cattle | α-S2-casein | 34.9 | 59.5 |
| 27881412 | Bos d 12 | Cattle | κ-casein | 37.9 | 56.3 |
| 30794348 | Bos d 9 | Cattle | α-S1-casein | 34.2 | 50.6 |
| 325910590 | Cav p 2 | Guinea Pig | Lipocalin | 42.4 | 65.7 |
| 33518896 | Cav p 4 | Guinea Pig | Albumin | 73.5 | 85.9 |
| 519113840 | Cav p 6 | Guinea Pig | Lipocalin | 48.5 | 68.9 |
| 520 | Bos d 5 | Cattle | β-lactoglobulin | 24.8 | 51.1 |
| 555980347 | Bos d 11 | Cattle | β-casein | 42.2 | 55.1 |
| 557943216 | Ory c 4 | Rabbit | Lipocalin | 63.2 | 82.2 |
| 868 | Can f 5 | Dog | Arginine esterase, prostatic kallikrein | 56.2 | 66.9 |
| 886209 | Bos d 3 | Cattle | S100 calcium-binding protein A7 | 35.0 | 56.0 |
| 886215 | Bos d 2 | Cattle | Lipocalin | 33.5 | 51.5 |

Supplemental table 2. Homologous T cell-reactive peptides from Mus m 1 and Mus m 1 isoforms.

| Mus m 1  peptide | Total SFC (donor average) | Mus m 1 isoforms peptide | Total SFC (donor average) |
| --- | --- | --- | --- |
| EWHTIILASDKREKI | 92 | EWFSILLASDKREKI | 332 |
| FRLFLEQIHVLENSL | 234 | FRLFLEQIRVLENSL | 314 |
| EQIHVLEKSLVLKFH | 128 | EQIRVLENSLVLKVH | 614 |
| LENSLVLKFHTVRDE | 116 | LENSLVLKVHTVRDE | 95 |
| GEYSVTYDGFNTFTI | 91 | GKYSVTYDGFNTFTI | 120 |
| TYDGFNTFTIPKTDY | 157 | NYDGFNTFSILKTDY | 30 |
| EPDLSSDIKERFAQL | 364 | EPYLSLDIKEKFAKL | 274 |
| CEEHGILRENIIDLS | 136 | CEKHGILRENIIDLS | 212 |
| ILRENIIDLSNANRC | 85 | IIRENIIDLTNVNRC | 164 |
| IIDLSNANRCLQARE | 172 | IIDLTNANRCLEARE | 8 |

Supplemental table 3. Peptide cluster analysis for T cell epitopes identified in Mus m 1 and Rat n 1.

| **Cluster** | **Peptide number** | **Peptide sequence** | **Allergen source** |
| --- | --- | --- | --- |
| A | 1 | TFQLMVLYGRTKDLSSDIKE | Rat n 1 |
| A | 2 | GLYGREPDLSSDIKERFA | Mus m 1 |
| A | 3 | YGREPDLSLDIKEK | Mus m 1 |
| A | 4 | GRTKDLSSDIKEKFAKLCEA | Rat n 1 |
| B | 1 | YDRYVMFHLINFKNGETFQL | Rat n 1 |
| B | 2 | AHLINEKDGETFQLM | Mus m 1 |
| B | 3 | LINFKNGETFQLMVLYGRTK | Rat n 1 |
| B | 4 | NEKDGETFQLMGLY | Mus m 1 |
| C | 1 | EENGSMRVFMQHIDVLENSL | Rat n 1 |
| C | 2 | GSMRVFVEHIHVLEN | Mus m 1 |
| D | 1 | FMQHIDVLENSLGFKFRIKE | Rat n 1 |
| D | 2 | FVEHIHVLENSLAFK | Mus m 1 |
| E | 1 | RDNIIDLTKTDRCLQARG | Rat n 1 |
| E | 2 | ENIIDLTKTNRCLKA | Mus m 1 |
| F | 1 | GDWFSIVVASNKREKIEENG | Rat n 1 |
| F | 2 | EWFSILLASDKREKI | Mus m 1 |
| G | 1 | EEASSTGRNFNVQKINGEWHTIIL | Mus m 1 |
| G | 2 | NVEKINGEWHTIIL | Mus m 1 |
| H | 1 | FVEYDGGNTFTILKTDYDRY | Rat n 1 |
| H | 2 | DGFNTFTILKTDYDN | Mus m 1 |
| I | 1 | TFTILKTDYDRYVMFHLINF | Rat n 1 |
| J | 1 | GIYYLNYDGFNTFTI | Mus m 1 |
| K | 1 | KTPEDGEYFVEYDGGNTFTI | Rat n 1 |
| L | 1 | LENSLVLKFHTVRDE | Mus m 1 |
| M | 1 | LQSGFYSLSSLVTVP | Mus m 1 |
| N | 1 | ENSLGFKFRIKENGECRELY | Rat n 1 |
| O | 1 | EKALVSSVRQRMKCS | Mus m 1 |
| P | 1 | LEQIHVLENSLVL | Mus m 1 |
| Q | 1 | DDVVASEALNSVWSGF | Mus m 1 |
| R | 1 | SRPFIFQEVIDLGGE | Mus m 1 |
| S | 1 | DKETLSLEELKALLL | Mus m 1 |
| T | 1 | IGGPDDGVITPWQSSF | Mus m 1 |
| U | 1 | DIKEKFAKLCEAHGITRDNI | Rat n 1 |
| V | 1 | RELYLVAYKTPEDGEYFVEY | Rat n 1 |
| W | 1 | ILGKLVKDYHLQFHR | Mus m 1 |
| X | 1 | TIFISLFLLSVCYSA | Mus m 1 |
| Y | 1 | EELRRLAPITSDPTE | Mus m 1 |
| Z | 1 | NLDVAKLNGDWFSIVVASNK | Rat n 1 |
| AA | 1 | LCEAHGITRDNIIDLTKTDR | Rat n 1 |
| AB | 1 | RIKENGECRELYLVAYKTPE | Rat n 1 |
| AC | 1 | ASNKREKIEENGSMRVFMQH | Rat n 1 |
| AD | 1 | EEASSTRGNLDVAKLNGDWF | Rat n 1 |
|  |  |  |  |

Supplemental table 4A. A full list of T cell-reactive peptides identified after redundancy elimination.

|  |  |  | **Average donor response (IL-5+IFNγ)** | |
| --- | --- | --- | --- | --- |
| **Peptide #** | **Protein** | **Sequence** | **Rhinitis (SFC)** | **Asthma (SFC)** |
| 1 | Mus m 1 | DNFLMAHLINEKDGE | 21 | 67 |
| 2 | Mus m 1 | ECSELSMVADKTEKA | 0 | 94 |
| 3 | Mus m 1 | EDNGNFRLFLEQIHV | 0 | 3 |
| 4 | Mus m 1 | EPDLSSDIKERFAQL | 185 | 390 |
| 5 | Mus m 1 | EQIHVLENSLVLKFH | 497 | 568 |
| 6 | Mus m 1 | IIDLSNANRCLQARE | 168 | 185 |
| 7 | Mus m 1 | ILASDKREKIEDNGN | 0 | 275 |
| 8 | Mus m 1 | KTEKAGEYSVTYDGF | 0 | 7 |
| 9 | Mus m 1 | LENSLVLKFHTVRDE | 90 | 125 |
| 10 | Mus m 1 | PKTDYDNFLMAHLIN | 10 | 0 |
| 11 | Mus m 1 | RFAQLCEEHGILREN | 11 | 6 |
| 12 | Mus m 1 | SDIKERFAKLCEEHG | 0 | 116 |
| 13 | Mus m 1 | SMVADKTEKAGEYSV | 0 | 61 |
| 14 | Mus m 1 | TYDGFNTFTIPKTDY | 14 | 168 |
| 15 | Mus m 1 | VLKFHTVRDEECSEL | 84 | 173 |
| 26 | Mup3 | SDEREKIEEHGSMR | 0 | 14 |
| 16 | Mup26 | IIRENIIDLTNVNRC | 160 | 214 |
| 17 | Mup26 | VFKFHFIVNEECTEM | 0 | 68 |
| 18 | Mup25 | DNYIMIHLINKKDGK | 0 | 242 |
| 19 | Mup25 | PLLLLLCLELTLVCI | 0 | 45 |
| 20 | Mup24 | ALKFHIIINEECSEI | 0 | 202 |
| 21 | Mup24 | LENSLALKFHIIINE | 0 | 163 |
| 22 | Mup13 | CEKHGILRENIIDLS | 162 | 244 |
| 23 | Mup11 | GKYSVTYDGFNTFTI | 0 | 129 |
| 24 | Mup1 | EWFSILLASDKREKI | 0 | 356 |
| 25 | Mup1 | MRVFVEHIHVLENSL | 167 | 200 |
| 27 | Mup 13 | EEASSTGRNFNVQKINGEWHTIIL | 0 | 160 |
| 28 | Mup 13 | GLYGREPDLSSDIKERFA | 125 | 355 |
| 29 | Mup 13 | LEQIHVLENSLVL | 230 | 475 |
| 30 | Mup 13 | NEKDGETFQLMGLY | 231 | 97 |
| 31 | Lysosomal thioesterase PPT2 | IGGPDDGVITPWQSSF | 0 | 7 |
| 32 | Leucine-rich HEV glycoprotein | GPTEFPSSLPADTV | 0 | 14 |
| 33 | Leucine-rich HEV glycoprotein | VGVATGSFQGLQHLDM | 0 | 17 |
| 34 | Lacrein | DDVVASEALNSVWSGF | 0 | 18 |
| 35 | Kidney androgen-regulated protein | LVSINKELQNSIIDLL | 0 | 4 |
| 36 | Kidney androgen-regulated protein | NKQNSEFSTDVETV | 0 | 9 |
| 47 | Kallikrein 1peptidase | DANWVLTAAHCYYEK | 10 | 0 |
| 48 | Kallikrein 1peptidase | DITDVVKPIALPTEE | 57 | 0 |
| 49 | Kallikrein 1peptidase | GGVLLDANWVLTAAH | 77 | 0 |
| 50 | Kallikrein 1peptidase | GVYTKLIKFTSWIKD | 276 | 197 |
| 51 | Kallikrein 1peptidase | LIKFTSWIKDTLAQN | 319 | 0 |
| 52 | Kallikrein 1peptidase | LMLLRLSKPADITDV | 87 | 0 |
| 37 | Ig kappa chain V-V | EAAIYHLQLFEELRR | 0 | 8 |
| 39 | Ig kappa chain V-V | HLQLFEELRRLAPIT | 0 | 6 |
| 40 | Ig kappa chain V-V | RSVEMLKEMIKSGMN | 0 | 14 |
| 41 | Ig kappa chain V-V | VDLRVNLAMDVGKAR | 0 | 5 |
| 38 | Ig kappa chain V-V | EELRRLAPITSDPTE | 0 | 6 |
| 43 | Ig kappa chain V-III MOPC 70 | EFDICFTSVQKRAIR | 0 | 4 |
| 44 | Ig kappa chain V-III MOPC 70 | FTSVQKRAIRTLWTV | 0 | 3 |
| 45 | Ig kappa chain V-III MOPC 70 | MAAYKLVLIRHGESA | 0 | 6 |
| 46 | Ig kappa chain V-III 50S10.1 | DIQMTQTTSSLSASL | 0 | 5 |
| 42 | Ig kappa chain V-III PC 7175 | VGDIMLIRDHINLPG | 0 | 62 |
| 53 | Alpha-S2-casein | LLHQYQKTMTPWSYY | 0 | 5 |
| 54 | Alpha-S2-casein | LTCLLAVALAKQRME | 0 | 8 |
| 55 | Alpha-S2-casein | MKFIILTCLLAVALA | 0 | 4 |
| 56 | Alpha-S2-casein | TMTPWSYYPSTPSQV | 0 | 18 |
| 57 | MUP 4 | DGKTFQLMELYGRKA | 241 | 43 |
| 58 | MUP 4 | IHVLENSLAFKFHTV | 160 | 206 |
| 59 | MUP 4 | VFVEHIHVLENSLAF | 242 | 272 |
| 60 | MUP 4 | YSVMYDGFNTFTILK | 0 | 151 |
| 61 | Odorant binding protein 1 | DWRTLYIAADKVEKV | 338 | 89 |
| 62 | Odorant binding protein 1 | MVKFLLIALALGVSC | 0 | 3 |
| 63 | Odorant binding protein 1 | QETNVILVAGKGETL | 34 | 4 |
| 64 | Alpha-S1-casein | LNLNQEFVNNMNRQR | 0 | 10 |
| 65 | Alpha-S1-casein | LTCLVAAAFAMPRLH | 0 | 8 |
| 66 | S100-A15A | DKETLSLEELKALLL | 0 | 7 |
| 67 | S100-A15A | FMDTLGRRQPYYITE | 0 | 14 |
| 68 | S100-A15A | ILGKLVKDYHLQFHR | 0 | 8 |
| 69 | S100-A15A | SLEELKALLLDSVPR | 0 | 22 |
| 70 | S100-A15A | VKDYHLQFHRQLCAH | 0 | 7 |
| 71 | MUP Iv Complex | ENIIDLTKTNRCLKA | 0 | 12 |
| 72 | MUP Iv Complex | FVEHIHVLENSLAFK | 350 | 264 |
| 73 | MUP Iv Complex | SVMYDGFNTFTILKT | 0 | 151 |
| 74 | BAC34145.1 | EKALVSSVRQRMKCS | 213 | 0 |
| 75 | BAC34145.1 | GERAFKAWAVARLSQ | 242 | 0 |
| 76 | BAC34145.1 | GFQNAILVRYTQKAP | 0 | 35 |
| 77 | BAC34145.1 | KLGEYGFQNAILVRY | 0 | 92 |
| 78 | BAC34145.1 | SVSLLLRLAKKYEAT | 135 | 0 |
| 79 | BAC34145.1 | TTFMGHYLHEVARRH | 210 | 4 |
| 80 | Vomeronasal secretory protein 1 | QYLAFEGKTHLQVQL | 0 | 5 |
| 81 | Odorant binding protein Ib | TKLLYILGKGEALTH | 0 | 9 |
| 82 | Odorant-binding protein 2a | EEFKKFAMSKGFREE | 0 | 95 |
| 83 | Odorant-binding protein 2a | FAMSKGFREENIIVP | 0 | 9 |
| 84 | MUP 5 | NTFTILKTDYDNYIM | 0 | 176 |
| 85 | Fab 7d11 Chain A | DSDGTYFLYSKLTVD | 0 | 10 |
| 86 | Fab 7d11 Chain A | LQSGFYSLSSLVTVP | 0 | 6 |
| 87 | Fab 7d11 Chain A | LWTTITIFISLFLLS | 0 | 2 |
| 88 | Fab 7d11 Chain A | SSGVRTVSSVLQSGF | 0 | 4 |
| 89 | Fab 7d11 Chain A | TIFISLFLLSVCYSA | 0 | 4 |
| 90 | Fab 7d11 Chain A | TVSSVLQSGFYSLSS | 0 | 12 |
| 91 | Fab 7d11 Chain A | VCYSASVTLFKVKWI | 0 | 5 |
| 92 | Fab 7d11 Chain A | VTVKWNYGALSSGVR | 0 | 5 |
| 93 | Cathepsin S | RRLIWEKNLKFIMIH | 0 | 12 |
| 94 | Cadherin 1 | RVEVPEDFGVGQEIT | 0 | 139 |
| 95 | Beta-globin | NDGLNHLDSLKGTF | 95 | 0 |
| 96 | Alpha-amylase | SRPFIFQEVIDLGGE | 150 | 0 |
| 97 | Equ c 4 | AINLEGLLNTILDQV | 0 | 14 |
| 98 | Equ c 4 | ELSVYLKLLILEPLT | 0 | 11 |
| 99 | Equ c 4 | KLLILEPLTLYVRTD | 0 | 16 |
| 100 | Equ c 4 | LLNTILDQVTGLLNI | 0 | 14 |
| 101 | Equ c 4 | LNSLVSNLDLQLVNN | 0 | 18 |
| 102 | Equ c 4 | LSVYLKLLILEPLTL | 0 | 21 |
| 103 | Equ c 4 | PLTLYVRTDIRVQLR | 0 | 20 |
| 104 | Equ c 4 | VTGLLNILVGPLLGP | 0 | 18 |
| 105 | Bos d 5 | YKKYLLVCMENSAEP | 71 | 0 |
| 106 | Procollagen, type I, alpha 2 | GPVGNPXGPAGPAGPRG | 0 | 3 |

Supplemental table 4B. A full list of T cell-reactive antigens identified.

|  |  | **Average donor response (IL-5+IFNγ)** | |
| --- | --- | --- | --- |
| **Antigen #** | **Protein** | **Rhinitis (SFC)** | **Asthma (SFC)** |
| 1 | Mus m 1 | 1081 | 2238 |
| 2 | Kallikrein 1 peptidase | 825 | 197 |
| 3 | BAC34145.1 | 801 | 131 |
| 4 | MUP 4 | 643 | 672 |
| 5 | MUP 13 | 586 | 1086 |
| 6 | Odorant binding protein 1 | 372 | 96 |
| 7 | MUP Iv Complex | 350 | 427 |
| 8 | Mup1 | 167 | 556 |
| 9 | Mup13 | 162 | 244 |
| 10 | Mup26 | 160 | 282 |
| 11 | Alpha-amylase | 150 | 0 |
| 12 | Beta-globin | 95 | 0 |
| 13 | Mup24 | 0 | 365 |
| 14 | Mup25 | 0 | 287 |
| 15 | MUP5 | 0 | 176 |
| 16 | Cadherin 1 | 0 | 139 |
| 17 | Mup11 | 0 | 129 |
| 18 | Odorant-binding protein 2a | 0 | 104 |
| 19 | Ig kappa chain V-III PC 7175 | 0 | 62 |
| 20 | S100-A15A | 0 | 58 |
| 21 | Fab 7d11 Chain A | 0 | 49 |
| 22 | Ig kappa chain V-V | 0 | 38 |
| 23 | Alpha-S2-casein | 0 | 34 |
| 24 | Leucine-rich HEV glycoprotein | 0 | 31 |
| 25 | Lacrein | 0 | 18 |
| 26 | Alpha-S1-casein | 0 | 17 |
| 27 | Mup3 | 0 | 14 |
| 28 | Ig kappa chain V-III MOPC 70 | 0 | 13 |
| 29 | Kidney androgen-regulated protein | 0 | 13 |
| 30 | Cathepsin S | 0 | 12 |
| 31 | Odorant binding protein Ib | 0 | 9 |
| 32 | Lysosomal thioesterase PPT2 | 0 | 7 |
| 33 | Ig kappa chain V-III 50S10.1 | 0 | 5 |
| 34 | Vomeronasal secretory protein 1 | 0 | 5 |
| 35 | Procollagen | 0 | 3 |

| **Donor ID** | **HLA-DPA1** | **HLA-DPA1** | **HLA-DPB1** | **HLA-DPB1** | **HLA-DQA1** | **HLA-DQA1** | **HLA-DQB1** | **HLA-DQB1** | **HLA-DRB1** | **HLA-DRB1** | **HLA-DRB3/4/5** | **HLA-DRB3/4/5** |
| --- | --- | --- | --- | --- | --- | --- | --- | --- | --- | --- | --- | --- |
| 1011 | N/A | N/A | DPB1*04:01 | DPB1*04:01 | N/A | N/A | DQB1*02:01 | DQB1*06:02 | DRB1*03:01 | DRB1*15:01 | DRB3*01:01 | DRB5*01:01 |
| 1435 | DPA1*02:01 | DPA1*01:03 | DPB1*17:01 | DPB1*141:01 | DQA1*01:01 | DQA1*03:03 | DQB1*02:02 | DQB1*05:01 | DRB1*01:02 | DRB1*09:01 | DRB4*01:01 | DRB4*01:01 |
| 1437 | N/A | N/A | DPB1*04:02 | DPB1*14:01 | N/A | N/A | DQB1*03:01 | DQB1*04:02 | DRB1*08:02 | DRB1*13:03 | DRB3*01:01 | N/A |
| 1440 | N/A | N/A | DPB1*01:01 | DPB1*04:01 | N/A | N/A | DQB1*02:02 | DQB1*03:01 | DRB1*07:01 | DRB1*12:01 | DRB3*02:02 | DRB4*01:01 |
| 1441 | N/A | N/A | DPB1*04:01 | DPB1*04:02 | N/A | N/A | DQB1*05:01 | DQB1*06:03 | DRB1*01:01 | DRB1*13:01 | DRB3*02:02 | N/A |
| 1460 | N/A | N/A | DPB1*02:01 | DPB1*04:01 | DQA1*01:02 | DQA1*02:01 | DQB1*02:02 | DQB1*06:02 | DRB1*07:01 | DRB1*15:01 | DRB4*01:01 | DRB5*01:01 |
| 1209 | DPA1*01:03 | DPA1*01:03 | DPB1*18:01 | DPB1*03:01 | DQA1*01:01 | DQA1*05:05 | DQB1*05:01 | DQB1*05:01 | DRB1*01:01 | DRB1*11:01 | DRB3*02:02 | N/A |
| 1277 | N/A | N/A | DPB1*01:01 | DPB1*02:01 | N/A | N/A | DQB1*06:02 | DQB1*05:02 | DRB1*11:01 | DRB1*15:03 | DRB3*03:01 | DRB5*01:01 |
| 1284 | N/A | N/A | DPB1*04:02 | DPB1*18:01 | N/A | N/A | DQB1*02:01 | DQB1*05:01 | DRB1*03:01 | DRB1*12:01 | DRB3*02:02 | DRB3*02:02 |
| 1368 | N/A | N/A | DPB1*01:01 | DPB1*01:01 | N/A | N/A | DQB1*02:02 | DQB1*05:01 | DRB1*01:01 | DRB1*09:01 | DRB4*01:01 | N/A |
| 1424 | N/A | N/A | DPB1*01:01 | DPB1*85:01 | N/A | N/A | DQB1*03:01 | DQB1*06:09 | DRB1*11:01 | DRB1*13:02 | DRB3*02:02 | DRB3*03:01 |
| 1425 | N/A | N/A | DPB1*13:01 | DPB1*19:01 | N/A | N/A | DQB1*05:01 | DQB1*06:03 | DRB1*12:01 | DRB1*13:01 | DRB3*01:01 | DRB3*01:01 |
| 1463 | N/A | N/A | DPB1*02:01 | DPB1*03:01 | N/A | N/A | DQB1*06:02 | DQB1*06:04 | DRB1*13:02 | DRB1*15:03 | DRB3*03:01 | DRB5*01:01 |
| 2017 | N/A | N/A | DPB1*02:02 | DPB1*05:01 | DQA1*01:03 | DQA1*03:01 | DQB1*04:01 | DQB1*06:01 | DRB1*04:05 | DRB1*08:03 | DRB4*01:01 | N/A |
| 1600 | N/A | N/A | DPB1*04:01 | DPB1*04:01 | N/A | N/A | DQB1*02:01 | DQB1*02:01 | DRB1*03:01 | DRB1*03:01 | DRB3*01:01 | DRB3*01:01 |
| 1704 | N/A | N/A | DPB1*02:01 | DPB1*04:01 | N/A | N/A | DQB1*03:01 | DQB1*06:03 | DRB1*04:01 | DRB1*13:01 | DRB3*01:01 | DRB4*01:01 |
| 1726 | N/A | N/A | DPB1*02:01 | DPB1*04:01 | N/A | N/A | DQB1*03:01 | DQB1*04:02 | DRB1*04:04 | DRB1*11:04 | DRB3*02:02 | DRB4*01:01 |
| 2397 | N/A | N/A | DPB1*02:01 | DPB1*03:01 | DQA1*03:01 | DQA1*03:01 | DQB1*03:01 | DQB1*03:02 | DRB1*04:01 | DRB1*04:01 | DRB4*01:01 | DRB4*01:01 |
| 2414 | N/A | N/A | DPB1*02:01 | DPB1*04:01 | DQA1*01:01 | DQA1*01:02 | DQB1*05:03 | DQB1*06:04 | DRB1*13:02 | DRB1*14:01 | DRB3*02:02 | DRB3*03:01 |
| 2423 | N/A | N/A | DPB1*03:01 | DPB1*03:01 | DQA1*03:01 | DQA1*05:01 | DQB1*02:01 | DQB1*03:02 | DRB1*03:01 | DRB1*04:04 | DRB3*02:02 | DRB4*01:01 |
| 2424 | N/A | N/A | DPB1*04:01 | DPB1*04:01 | DQA1*01:03 | DQA1*03:01 | DQB1*03:02 | DQB1*06:03 | DRB1*04:05 | DRB1*13:01 | DRB3*02:02 | DRB4*01:01 |
| 2489 | N/A | N/A | DPB1*02:01 | DPB1*05:01 | DQA1*01:01 | DQA1*06:01 | DQB1*03:01 | DQB1*05:03 | DRB1*12:02 | DRB1*14:05 | DRB3*02:02 | DRB3*03:01 |
|  |  |  |  |  |  |  |  |  |  |  |  |  |
|  |  | N/A- not applicable |  |  |  |  |  |  |  |  |  |  |

Supplemental table 5. A full list of HLA types expressed in the donor cohort.

Supplemental table 6. CD154 expression and percent cytokine production after short- term ex vivo stimulation.

|  |  |  |  | **Within CD154+ population** | | | |
| --- | --- | --- | --- | --- | --- | --- | --- |
| **Cohort** | **Donor** | **Stimulus** | **% CD154+** | **% IL-4+** | **% IL-10+** | **% IL-17+** | **% INFg+** |
| Asthmatic | 1726 | Medium | 2.61 | 1.75 | 0.44 | 0.88 | 1.97 |
|  | 2397 |  | 1.92 | 0.00 | 1.56 | 2.34 | 0.00 |
|  | 1440 |  | 0.73 | 2.22 | 0.00 | 2.22 | 8.89 |
|  | 1463 |  | 0.75 | 0.00 | 0.00 | 0.00 | 0.00 |
|  | 1435 |  | 2.27 | 0.00 | 0.00 | 0.00 | 1.43 |
|  | 1011 |  | 2.35 | 0.00 | 0.73 | 2.19 | 0.00 |
| Rhinitic | 2414 |  | 2.98 | 0.00 | 0.00 | 0.68 | 0.34 |
|  | 2423 |  | 0.99 | 0.95 | 0.00 | 0.00 | 3.81 |
|  | 2424 |  | 1.63 | 0.47 | 0.00 | 1.42 | 0.95 |
|  | 2489 |  | 0.79 | 0.00 | 0.00 | 2.50 | 0.00 |
|  | 1460 |  | 0.85 | 1.59 | 0.00 | 7.94 | 3.17 |
|  | 1704 |  | 1.45 | 0.98 | 0.98 | 0.00 | 0.98 |
| Asthmatic | 1726 | Megapool | 7.40 | 0.80 | 0.00 | 0.80 | 4.42 |
|  | 2397 |  | 5.33 | 1.60 | 0.00 | 0.00 | 4.26 |
|  | 1440 |  | 1.52 | 0.00 | 0.00 | 3.85 | 3.85 |
|  | 1463 |  | 1.57 | 0.00 | 0.00 | 0.00 | 0.00 |
|  | 1435 |  | 6.10 | 0.00 | 0.00 | 0.00 | 1.20 |
|  | 1011 |  | 15.20 | 9.77 | 0.00 | 0.93 | 7.44 |
| Rhinitic | 2414 |  | 1.74 | 1.94 | 0.00 | 0.97 | 1.94 |
|  | 2423 |  | 2.23 | 1.23 | 0.00 | 0.31 | 6.75 |
|  | 2424 |  | 5.01 | 2.39 | 0.80 | 2.39 | 6.37 |
|  | 2489 |  | 10.60 | 36.40 | 0.78 | 1.16 | 3.10 |
|  | 1460 |  | 2.23 | 2.13 | 0.00 | 0.00 | 11.70 |
|  | 1704 |  | 3.34 | 0.00 | 0.00 | 0.00 | 11.00 |
| Asthmatic | 1726 | Epithelial extract | 1.73 | 2.78 | 0.40 | 1.59 | 1.59 |
|  | 2397 |  | 1.19 | 0.00 | 0.00 | 0.00 | 2.82 |
|  | 1440 |  | 0.63 | 4.55 | 0.00 | 0.00 | 13.60 |
|  | 1463 |  | 2.90 | 3.85 | 0.00 | 3.85 | 23.10 |
|  | 1435 |  | 1.74 | 0.00 | 0.00 | 5.00 | 7.50 |
|  | 1011 |  | 1.50 | 12.60 | 0.00 | 0.00 | 1.15 |
| Rhinitic | 2414 |  | 3.36 | 2.15 | 0.00 | 0.36 | 1.43 |
|  | 2423 |  | 1.09 | 1.41 | 0.00 | 2.82 | 0.00 |
|  | 2424 |  | 1.48 | 1.45 | 0.00 | 2.90 | 3.62 |
|  | 2489 |  | 2.19 | 27.40 | 0.00 | 1.61 | 0.00 |
|  | 1460 |  | 1.66 | 3.57 | 0.00 | 0.00 | 17.90 |
|  | 1704 |  | 0.50 | 0.00 | 0.00 | 0.00 | 0.00 |
| Asthmatic | 1726 | Urine | 2.68 | 1.63 | 0.54 | 2.72 | 0.82 |
|  | 2397 |  | 0.65 | 0.00 | 0.00 | 0.00 | 2.08 |
|  | 1440 |  | 0.47 | 0.00 | 0.00 | 0.00 | 5.00 |
|  | 1463 |  | 1.01 | 3.70 | 0.00 | 3.70 | 22.20 |
|  | 1435 |  | 1.68 | 1.59 | 1.59 | 3.17 | 11.10 |
|  | 1011 |  | 3.91 | 22.70 | 0.00 | 0.00 | 2.13 |
| Rhinitic | 2414 |  | 2.70 | 2.70 | 0.00 | 0.00 | 0.77 |
|  | 2423 |  | 0.93 | 0.99 | 0.00 | 0.99 | 3.96 |
|  | 2424 |  | 1.67 | 2.45 | 0.49 | 4.90 | 0.98 |
|  | 2489 |  | 2.28 | 28.30 | 0.00 | 0.00 | 1.09 |
|  | 1460 |  | 0.58 | 0.00 | 0.00 | 6.25 | 0.00 |
|  | 1704 |  | 2.32 | 3.30 | 0.00 | 1.10 | 7.69 |
| Non-allergics | 1774 | Megapool | 3.74 | 0.44 | 0.44 | 0.00 | 7.46 |
|  | 2015 |  | 2.64 | 0.00 | 0.00 | 2.01 | 8.05 |
|  | 2458 |  | 2.15 | 0.00 | 0.00 | 1.18 | 1.18 |
|  | 2491 |  | 1.56 | 0.00 | 0.00 | 0.00 | 4.13 |
|  | 2500 |  | 0.16 | 0.00 | 20.00 | 0.00 | 0.00 |
|  | 2501 |  | 5.23 | 0.00 | 0.49 | 2.94 | 0.98 |
|  | 2503 |  | 9.22 | 4.10 | 0.00 | 0.00 | 2.46 |
|  | 2544 |  | 1.83 | 0.00 | 0.61 | 0.61 | 6.75 |
|  | 2547 |  | 4.80 | 0.55 | 0.00 | 0.00 | 1.65 |
|  | 2555 |  | 3.51 | 0.00 | 0.00 | 0.00 | 4.07 |
